# Quantitative proteomics of rat livers shows that unrestricted feeding is stressful for proteostasis with implications on life span

**DOI:** 10.18632/aging.101009

**Published:** 2016-08-09

**Authors:** Galia Gat-Yablonski, Andrija Finka, Galit Pinto, Manfredo Quadroni, Biana Shtaif, Pierre Goloubinoff

**Affiliations:** ^1^ The Jesse Z and Sara Lea Shafer Institute for Endocrinology and Diabetes, National Center for Childhood Diabetes, Schneider Children's Medical Center, Petach Tikva, Israel; ^2^ Felsenstein Medical Research Center, Petach Tikva, Israel; ^3^ Sackler School of Medicine, Tel Aviv University, Ramat Aviv, Israel; ^4^ Department of Plant Molecular Biology, Faculty of Biology and Medicine, University of Lausanne, 1015 Lausanne, Switzerland; ^5^ Protein Analysis Facility, University of Lausanne, 1015 Lausanne, Switzerland; ^6^ Department of Ecology, Agronomy and Aquaculture, University of Zadar, 23000 Zadar, Croatia

**Keywords:** heat shock proteins, catch up growth, food restriction, aging, mitochondria

## Abstract

Studies in young mammals on the molecular effects of food restriction leading to prolong adult life are scares. Here, we used high-throughput quantitative proteomic analysis of whole rat livers to address the molecular basis for growth arrest and the apparent life-prolonging phenotype of the food restriction regimen. Over 1800 common proteins were significantly quantified in livers of ad libitum, restriction- and re-fed rats, which summed up into 92% of the total protein mass of the cells. Compared to restriction, ad libitum cells contained significantly less mitochondrial catabolic enzymes and more cytosolic and ER HSP90 and HSP70 chaperones, which are hallmarks of heat- and chemically-stressed tissues. Following re-feeding, levels of HSPs nearly reached ad libitum levels. The quantitative and qualitative protein values indicated that the restriction regimen was a least stressful condition that used minimal amounts of HSP-chaperones to maintain optimal protein homeostasis and sustain optimal life span. In contrast, the elevated levels of HSP-chaperones in ad libitum tissues were characteristic of a chronic stress, which in the long term could lead to early aging and shorter life span.

## INTRODUCTION

Children malnutrition, marked by various nutrient deficiencies, is considered a leading cause for growth attenuation and failure to thrive. While in children it is difficult to dissociate the effect of nutritional factors from other environmental cues and to ascertain the irreversibility of a severe nutritional damage [1, 2], studies in young animals, focusing on the epiphyseal growth plate (EGP) [3-5], demonstrated the deleterious effects of food restriction (RES) on linear growth. On the other hand, mild continuous RES in adults is the most accredited treatment against aging [7]. In mice, a protocol of 40% RES was shown to cause a significant 150% increase in mean life span (27.4 ± 0.9 months of AL vs 42.3±0.9 months for RES mice) [6, 7], by way of yet poorly understood molecular and cellular mechanisms. In young mammals exposed to a period of RES, re-feeding usually leads to catch-up (CU) growth. This phenomenon, defined as “height velocity above the normal statistical limits for age and/or maturity during a defined period of time, following a transient period of growth inhibition” [8], is common in children during longitudinal growth, after the removal of a growth inhibiting condition. It usually culminates with the organism attaining a similar position on the growth curve as without food restriction. However, CU in youth is associated with increased propensities to develop metabolic complications in late adulthood. Hence, adults that undergo successful CU growth in childhood have increased risk to develop metabolic syndromes such as obesity, dyslipidemia, hypertension, insulin resistance and diabetes [9-12]. In contrast, a continuous mild RES regimen in young adult animals and humans, in which linear growth is minimally affected, is associated with the attenuation of a large spectrum of age-associated diseases, such as diabetes and immune dysfunction [13], leading to an apparent increase of life span [6, 7].

In animals, the time of natural death is determined by the duration and the severity of a preceding period of aging, during which the protein quality control machineries of cells, which are predominantly composed of heat shock protein (HSP) acting as unfolding and disaggregating chaperones and by chaperone-gated proteases, become defective. HSP-chaperone failure in aging leads to the accumulation of cytotoxic protein aggregates that cause untimely cell death, tissue loss and the early onset of degenerative protein misfolding disorders, such as amyotrophic lateral sclerosis, Parkinson's and Alzheimer's diseases [14].

A large body of experimental evidence in human and model organisms, such as *C. elegans*, flies and yeast, indicate that aging is the consequence of increased intra- and extra-cellular levels of misfolded protein aggregates, which are cytotoxic. Hence, the primary cause for the onset in late age of neural tissue loss in Parkinson and Alzheimer's diseases is the apparent stress- chemically- and/or mutation-induced accumulation of toxic misfolded alfa-synuclein, or tangles of the tau protein in- and outside cells. Such aggregates may condense into protofibrils and compact fibrils that can directly damage membranes and indirectly produce harmful reactive oxygen species that amplify and mediate the detrimental apoptosis signals, leading to progressive tissue degeneration [15-17]. Noticeably, RES delays the age of onset of protein misfolding disorders in organisms as diverse as yeast, flies, nematodes and mammals [18, 19]. RES decreases the formation of ROSs in mitochondria [20]. It also increases mitochondrial biogenesis, preserves intestinal stem cells [21], enhances stem cell function in skeletal muscles [22] and reduces pathology in mouse models of Alzheimer's disease [23] and of amyotrophic lateral sclerosis [24].

We have previously established a model to investigate the mechanism underlying the nutritional-growth-metabolism connection, wherein young rodents were subjected to 40 % RES for 10 days, followed by one day of unrestricted re-feeding (CU) [11]. During the experiment, RES rats gained only 0.81 g/day, whereas *ad libitum* (AL) fed rats gained 6.4 g/day. When food restriction was removed (CU), the rats rapidly gained weight, with a dramatic weight increase of around 17.3 g in a single day. In addition, while EGP height was significantly reduced in RES rats, it was nearly fully regained within one day of CU. Internal organs including liver, heart, kidneys and lungs showed a similar rapid weight regain response (Table 1) [11].

**Table 1:** Body weight, liver weight and crude liver fat content

Sample	Body weight (Baseline) (g)	Body weight (Termination) (g)	Liver weight (BC) (g)	Liver weight (Termination)(g)	Fat (mg/100 mg Liver)
AL	57.5±7.8^a^	127.6±12.3 ^a^	2.45±0.18	6.2±1.1 ^a^	6.3±1.8 ^a^
RES	60.7±9.1 ^a^	69.3±3.9 ^b^		2.75±0.45 ^b^	6.3±0.6 ^a^
CU	54.9±9.3 ^a^	86.58±5.8 ^c^		4.6±0.3 ^c^	7.1±1.5 ^a^

Comparisons were done within each column. Superscripts marked with the same letter do not differ significantly at P<0.05.

Here, we used mass spectrometry (MS)-based label-free quantitative proteomic analysis of whole livers from young rats, in an attempt to understand how RES might lead to growth attenuation while apparently prolonging adult life span, and to identify the major qualitative and quantitative changes in the protein profiles of RES and CU or AL livers. We found that although the very slow growing RES liver cells massively accumulated specific mitochondrial proteins [25-27], they did not necessitate high levels of HSP chaperones to carry out optimal folding and warrant optimal life span. In contrast, the massive protein synthesis in rapidly growing AL cells was found to recruit high levels of ribosomes and HSP chaperones, in particular cytosolic Hsp90AB1 and HSC70 (HSPA8) and endoplasmic reticulum HSP90B1 and BIP (HSPA5), which are general hallmarks of heat-, chemical- and/or cellular-stresses [28, 29]. Proteomic values showed that although massive protein synthesis is required for rapid growth of the young animals, when sustained, this brings upon a continuous strain on the protein quality control machineries of AL and CU liver cells, which could lead in the long term to the formation of toxic protein aggregates ultimately reducing life span.

## RESULTS

**Figure 1:**
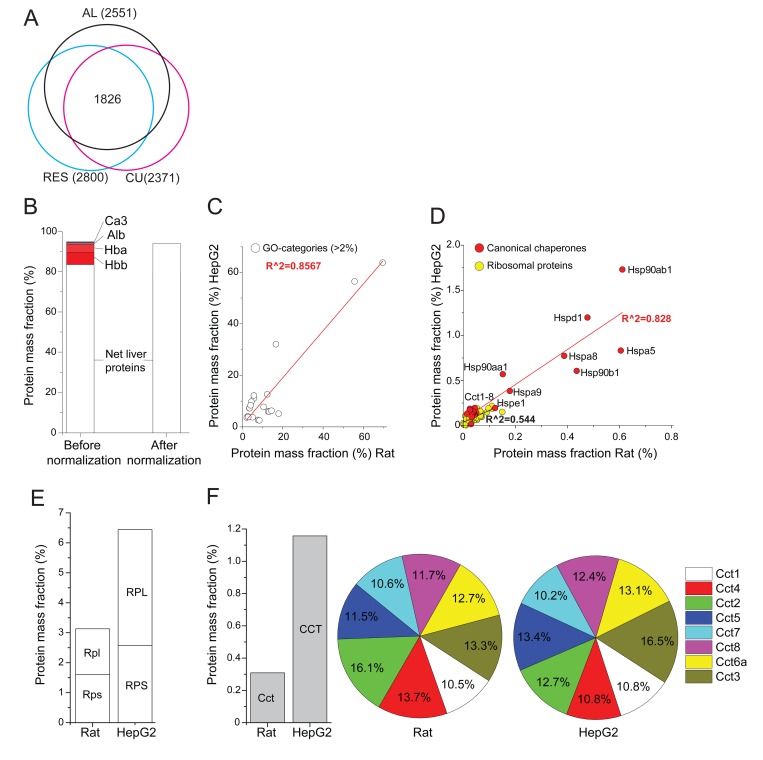
Relative mass fraction values of significantly quantified proteins from rat livers (**A**) Venn diagram: numbers (in brackets) of significantly quantified proteins, separately in AL-, RE- and CU livers. The sum of masses of the 1826 significantly quantified proteins in all three treatments was 92% of the total mass. (**B**) The sum of the relative mass fractions of the 2551 significantly quantified proteins in AL livers, before (left) or after (right) their correction by subtracting the most abundant blood proteins, Hemoglobin (Hba, Hbb), carbonic anhydrase (Ca3) and serum albumin (Alb) and expressing them as net liver cell values. (**C**) Correlation between the total relative masses of the 21 most abundant gene ontology (GO) categories that contain more than 2% of the total protein mass in AL rat liver cells, as compared to published human HepG2 cancer cells (see Supplementary Table 2). (**D**) Correlation between the total relative masses of core chaperones (red) and ribosomal proteins (yellow) in AL rat liver cells, as compared to HepG2 cells. (**E**) Sum of relative mass proportions (%) of the large and small ribosomal proteins in AL rat liver cells (left), as compared to HepG2 cells (right). (**F**) Sum of relative masses (%) in AL rat liver and HepG2 cells of all CCT chaperonins (grey). (**G**) Relative mass distribution among the eight homologous subunits CCT_1-8_ (each differentially colored) in the hetero-hexadecameric chaperonin complex (right).

For the six AL samples, the analysis of the 5138 identified proteins yielded 2551 proteins with average means that minimally varied bellow the statistical threshold of p<0.05. The sum of their relative masses was 95 % of the total protein mass of the cells, implying that only 5 % of the proteins among the six different biological AL samples, varied excessively, above our chosen statistical threshold. Unless specified otherwise, proteins with excessively variable mass fractions were not included in the analysis. Similarly, 2800 proteins in RES and 2371 proteins in CU were found to have statistically significant values. In each case, they summed up into being more than 95 % of the total protein mass (Supplementary Table 1, Sheet 2). Noticeably, of those, 1826 were present and reliably quantified in all three treatments, which accounted for 92 % of the total protein mass in all 18 liver samples (Fig. 1A). It should be noted that the 18 liver samples were derived from 18 distinct animals. Since the rats were not from a highly inbred strain, a certain degree of natural variability can be anticipated.

The most abundant protein in the six AL samples was mitochondrial carbamoyl-phosphate synthase 1 (CPS1), which accounted for 7.8 % of the total liver proteins. The next most abundant proteins were the two main hemoglobins (Hba and Hbb, 10.8 %), indicating that the liver tissues contained blood proteins (serum and erythrocytes) (Fig. 1B, before normalization). We next pooled the major blood proteins, Hba, Hbb, serum albumin and carbonic anhydrase, which in AL summed up to be 12.7 %, and excluded them from subsequent calculations of the net liver cell proteins. This produced in AL, 2547 significantly quantified non-blood proteins, whose relative masses summed up to be 96.3 % (Supplementary Table 1, Sheet 4) of the net total protein mass of liver cells (Fig. 1B, after normalization).

We next addressed the general functions and amounts of various groups of proteins in rat liver cells, as compared to previously published quantitative data from cultured human HepG2 hepatocyte cells. The 2547 significantly quantified proteins from rat naïve liver cells (this study) and 2428 from the HepG2 cancer cells (from [34]) were separately sorted according to gene ontology (GO) categories and analyzed for their statistically significant representation in the sample (p<0.05), using Panther DB web server (www.pantherdb.org). The mass fraction of the proteins in each category was summed up and 21 GO categories were retained for containing each at least 2% of the total protein mass of the cells. The relative sums of masses of each of the 21 GO categories correlated very well (R^2^=0.86) between the two cell types, in particular protein, nitrogen, lipid, fatty acid and carbohydrate metabolisms (Supplementary Table 2, sheet 1). Thus, in general, rat liver cells and the HepG2 cells that originate from human liver cancer, coherently expressed to similar levels, similar sets of abundant proteins (Fig. 1C).

Examination of the relative mass fractions of 73 commonly detected cytosolic ribosomal proteins suggested an estimated density of ribosomes for naïve rat liver cells that was about twice lower than in HepG2 cells (Fig. 1D; Supplementary Table 2). The average volume and total protein mass of a typical average rat liver cell being 8354 μm^3^ and 985 pg, respectively [35] (Supplementary Table 2), and knowing the individual molecular weights of the quantified polypeptides, the mass fraction of each ribosomal protein was thus converted into copy number per μm^3^ of liver cell. This produced a median value of 1400 ribosome particles per μm^3^ of AL cell, a ribosomal density that matched very well with recent estimates in human Jurkat cells of 2000 particles per μm^3^ [29]. This value also matched earlier estimates based on independent rRNA quantitation in human cells, in which ribosomes contributed between 5–10 % to the total protein mass [36).

### Chaperone distribution and amounts

A category that mass-wise markedly differed between rat hepatocytes and human HepG2 cells was the main core chaperones [28, 37, 38) (Fig 1E). The cytosolic HSP90AA1, HSP90AB1, HSPA8 (HSC70) and TCP1&CCT2-8, the ER HSP90B1 (endoplasmin) and HSPA5 (Bip) were two to threefold more abundant, and mitochondrial HSPD1 (HSP60) and HSPA9 (mortalin) were nearly tenfold more abundant in HepG2 cells than in naïve liver cells (Supplementary Fig. 1). Together, they summed up into being 1.6% of the total rat liver proteins, as compared to 7.7% of the total HepG2 proteins. This significant difference was expected, as HepG2 cells are cancer cells, for the survival of which, an effective massive accumulation of HSP chaperones is essential [39]. This specific constitutive over-expression of molecular chaperones was exemplified in the case of the eight homologous cytosolic CCT chaperonin subunits, which in HepG2 cells were about four times more abundant than in the rat liver cells (Fig. 1F, left). Despite this dramatic difference, the relative equistoichiometric distribution of the eight different CCT subunits in the chaperonin hetero-hexadecamers was maintained (Fig. 1F, Fig. 1G). The very minor variation of the measured relative masses for each of the eight CCT subunits, which were found to be close to their theoretical mean of 12.5 %, confirms that the method to calculate protein amounts from MS data is valid and accurate.

### Significant variations of particular proteins between AL, RES and CU

During the eleven days of the experiment, the total body and liver masses of RES rats increased merely 1.14 times compared to AL rats that increased ∼2.2 times (Table 1). It should be noted that the net caloric balance of the RES regime was slightly positive given that the body weight and RES livers did not shrink but rather allowed a minimal net growth of about 14%. A significant proportional increase of total crude lipid (Table 1) and also of glycogen content [11], were observed in AL, as compared to RES, implying that twice more newly produced proteins accumulated in rapidly growing AL tissues, as compared to the slow growing RES tissues.

**Figure 2:**
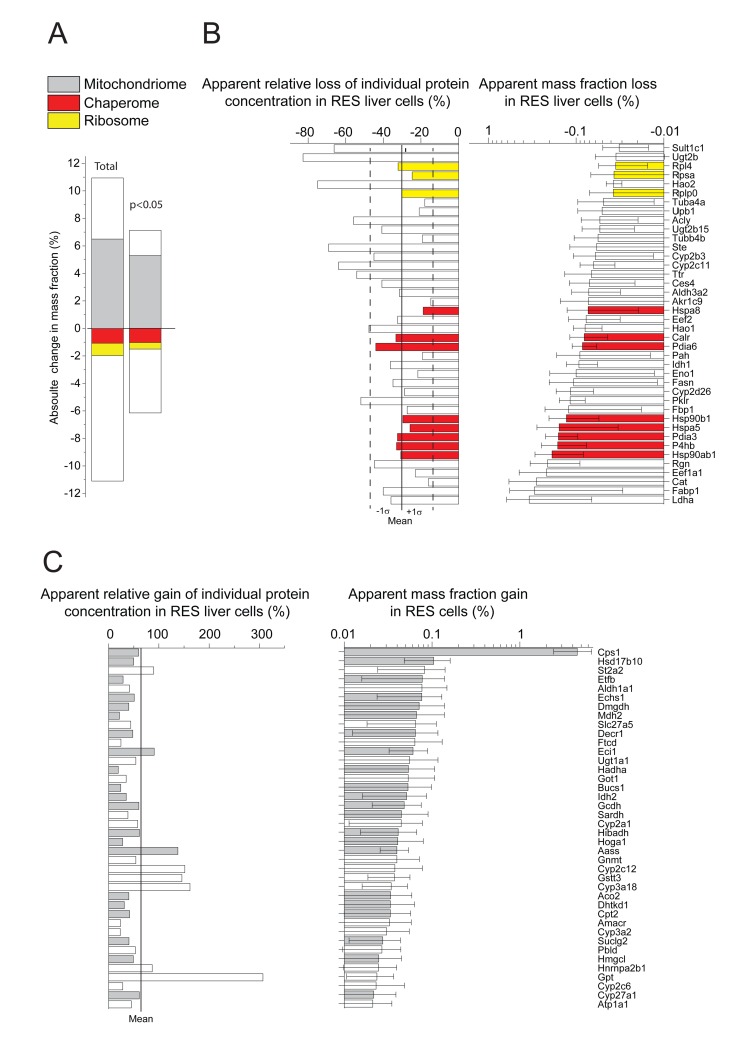
Net significant differences in protein masses between RES and AL (**A**) Sum of relative mass gains and losses in RES compared to AL. Right: Only the proteins with significant (P<0.05) mass differences Left: All detected proteins. (**B**) Relative (left) and absolute (right) mass losses of the 40 most depleted proteins in RES compared to AL. (**C**) Relative (left) and absolute (right) mass gains of the 40 most accumulated proteins in RES compared to AL. Grey, mitochondrial proteins. Red: chaperome proteins. Yellow: ribosomal proteins. White: others.

Whereas most proteins maintained equal proportion in both AL and RES cells, some proteins, many of which mitochondrial enzymes, that summed up to be 11 % of the total proteins, were significantly more abundant in RES than in AL cells. Conversely, other proteins, most of which ribosomal proteins and HSP-chaperones, that also summed up to be 11 % of the total proteins, were significantly less abundant in RES than in AL cells (Fig. 2A, left). When considering only the 617 proteins for which the individual masses were found to significantly differ (P<0.05) between the two regimens, 266 were more abundant in RES than in AL and summed up to be 7 % of the total protein mass, and 351 proteins were less abundant in RES than in AL and summed up to be 6.2 % of the total protein mass (Fig. 2A, right; Supplementary Table 3). Noticeably, when considering both at high (P<0.05) and low levels of statistical significance (P>0.05), there was in RES a strong bias for more mitochondrial catabolic enzymes and for less cytosolic ribosomal and HSP chaperones, than in AL. Specifically, the cytosolic HSPA8 (HSC70), HSP90B1 and the endoplasmic reticulum HSPA5 (Bip) and HSP90AB1 (GRP94), which are among the most abundant proteins e.g. in human cells and may further accumulate under heat-shock [29]), were among the 40 proteins that were most decreased, on average 30 %, in RES as compared to AL (Fig. 2B).

### Changes in metabolic enzymes

Results indicated that when growth was very slow in RES, the protein synthesis, which was also much slower than in AL, was mostly recruited to synthesize mitochondrial catabolic enzymes, to induce mitochondrial biogenesis and increase bioenergetic efficiency [20]. Noticeably, under all conditions, liver mitochondria contained very high amounts of Carbamoyal-Phosphate-Synthase (CPS1), which catalyzes the first committed step to convert the highly toxic ammonia, produced by amino acid catabolism, into nontoxic urea. Whereas CPS1 was 12 % of the total protein mass in RES liver cells, it was dramatically less, 7.5 %, in AL liver cells. The massive amounts of CPS1 in RES and its much lower levels in AL were confirmed by SDS-PAGE and Coomassie staining of total protein extracts from the AL, RES and CU livers (Supplementary Fig. 2).

**Figure 3:**
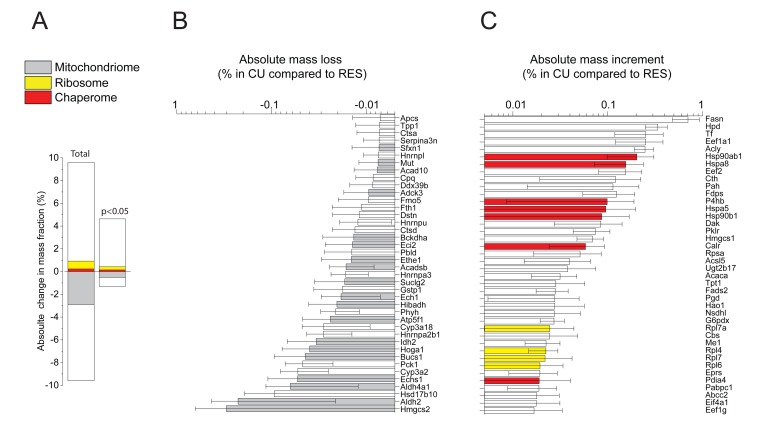
Net changes in protein masses between CU and RES (**A**) Sum of relative mass gains and losses in CU as compared to RES. Left: only proteins with significant (P<0.05) mass differences. Right: All detected proteins. (**B**) Net absolute mass losses of the 40 most depleted proteins in CU. (**C**) Net absolute mass gains of the 40 most accumulated proteins in CU. Grey, mitochondrial proteins. Red: chaperome proteins. Yellow: ribosomal proteins. White: others.

When, following ten days of RES regimen, a single day of unlimited re-feeding (CU) was applied, this resulted in a dramatic weight increase of the liver, initially from ∼50%, and reaching ~75% of the AL values (Table 1). The relative fatty acid (FA) content per 100 mg liver tissue was similar in all conditions, implying that during the single day of CU, the absolute FA content per liver nearly doubled (Table 1). The quantitative proteomic analysis revealed that the CU livers underwent in 24h a general qualitative and quantitative change of their initial RES protein profile, towards an AL-like protein profile. The total mass differences, when including also values with low statistical significance, indicated that compared to RES, up to 9.5 % of the proteins became either more abundant or reciprocally less abundant in CU (Fig. 3A, left). When computing the 512 proteins whose individual masses were found to significantly differ between CU and RES, 390 were more abundant in CU than in RES, which summed up to be 4.7 % of the total protein mass, and 122 proteins were less abundant, which summed up to be 1.6 % of the total protein mass (Fig. 3A, right; Supplementary Table 3). Noticeably, at both levels of significance, many mitochondrial proteins were apparently degraded as they became abruptly less abundant (Fig. 3B) and many ribosomal and chaperone proteins became significantly more abundant in CU than in RES (Fig. 3C), implying that the latter were massively and preferably synthetized during the single day of re-feeding.

**Figure 4:**
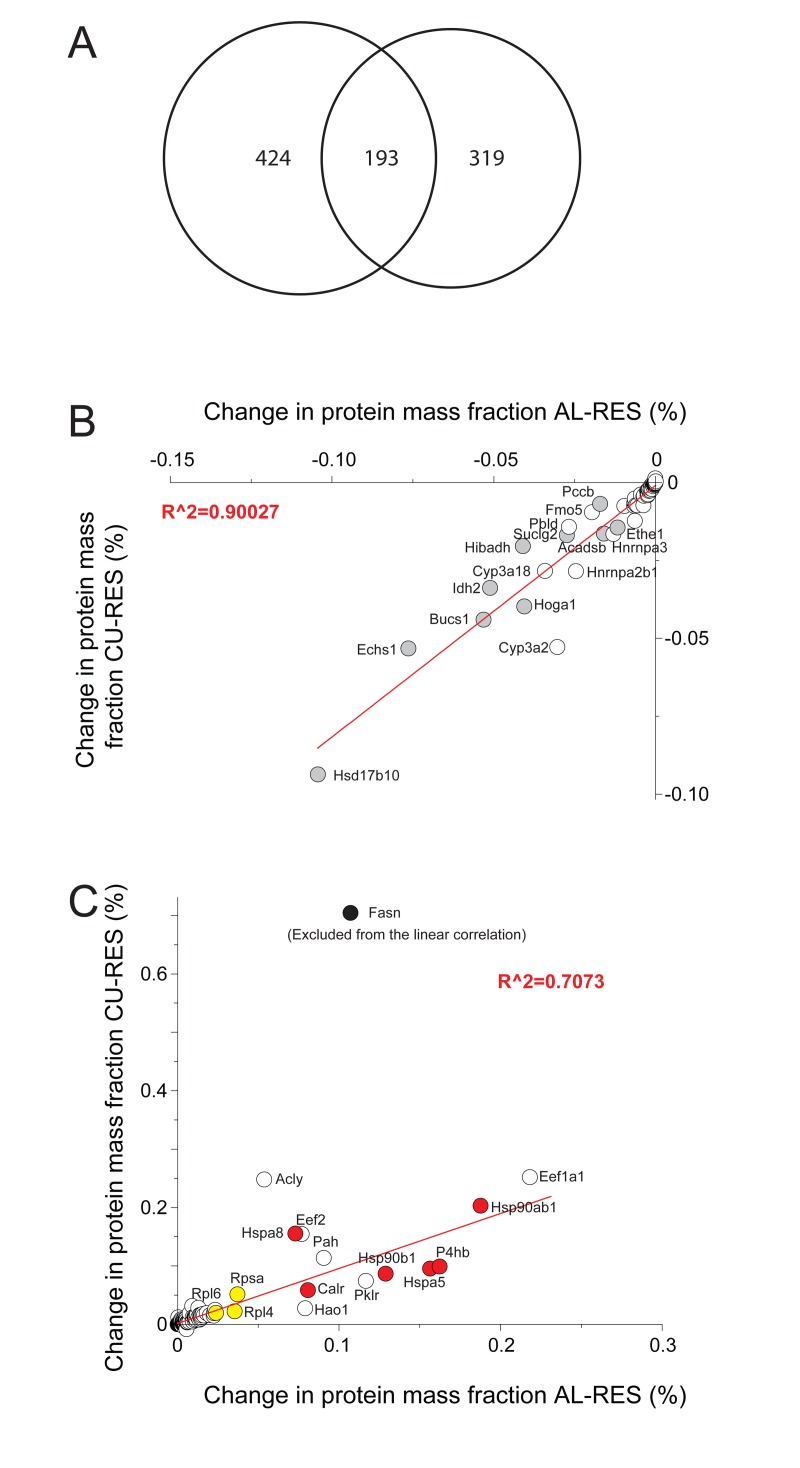
Differences in protein masses between RES, AL and CU regimes **(A**) Venn diagram showing the 617 proteins, whose mass was significantly different between RES and AL (left) and the 513 proteins whose mass became significantly changed during one day of RES to CU transition (right). 193 proteins (center) varied significantly in both comparisons. (**B**) Proteins whose mass proportions were significantly higher in RES than in AL and CU. (**C**) Proteins whose mass proportions were significantly lower in RES than in AL and CU. Grey, mitochondrial proteins. Red: chaperone proteins. Yellow: ribosomal proteins. White: others.

Amounts of 193 proteins were found to significantly differ between the RES and AL and RES and CU treatments (Fig. 4A). Their individual mass fractions varied anti-correlatively: within one day of CU, proteins such as CPS1 and many other catabolic enzymes of the mitochondria that were more abundant in RES, were found to be either more rapidly degraded or more slowly synthesized than in AL and CU. Reciprocally, proteins such as cytosolic ribosomal proteins and HSP-chaperones and ER chaperones, which were proportionally less abundant in RES, were significantly more abundant in the AL and CU samples (Fig. 4B and C). Thus, protein-wise, a single day of re-feeding sufficed to call off the mitochondrial catabolic and respiratory functions that were particularly active during the ten days of RES.

The near complete growth arrest during RES suggests that most of the limited food resources were allocated to energy production by the mitochondria. This indicates that the amino acids, which were generated by protein degradation in RES, were not used to synthesize new proteins, but were rather degraded into carbon chains available for oxidative respiration by the mitochondria, and ammonia, which were efficiently detoxified into urea by the increased amount of CPS1. Hence, in RES, most of the amino acids that became incorporated into newly synthesized mitochondrial proteins likely came from the limited protein pool within the RES food. As soon as food resources became unlimited as in AL, the mitochondria-oriented program was readily called off. Cells in the AL and CU livers were rapidly proliferating, as suggested by the observed increase in PCNA (Table 2; Supplementary Fig. 3) as well as by the massive synthe-sis of new proteins which expectedly necessitated more ribosomes and more HSP chaperones to mediate proper protein folding, translocation and assembly (Table 2).

**Table 2:** Measured cellular concentrations of key metabolic enzymes, components of the cellular proteostasis machineries, proteins involved in growth regulation, glycogen synthesis and lipid catabolism

Protein names	copy number (AL)	copy number (RES)	copy number (AL-RES)	T-test (AL-RES)*	copy number (CU)	copy number (CU-RES)	T-test (CU-RES)
Cps1, Carbamoyl-phosphate synthase (M)	44125	70441	−26316	0.001	60291	−10150	0.087
Etfb, Electron transfer flavoprotein subunit beta (M)	9357	12041	−2684	0.015	11318	−723	0.433
Hsd17b10, 3-hydroxyacyl-CoA dehydrogenase type-2 (M)	7431	11123	−3692	0.001	7808	−3315	0.057
Mdh2, Malate dehydrogenase (M)	8402	10195	−1793	0.048	10160	−34	0.984
Echs1, Enoyl-CoA hydratase (M)	4593	6930	−2337	0.006	5301	−1629	0.042
HSPA5, 78 kDa glucose-regulated protein, GRP78, BiP, (ER)	8050	5968	2082	0.013	7240	1272	0.045
St2a2, Alcohol sulfotransferase A (C)	2661	5046	−2385	0.006	4903	−143	0.888
Aldh1a1, Retinal dehydrogenase 1 (C)	3273	4629	−1356	0.028	4470	−159	0.826
Got1, Aspartate aminotransferase, (C)	3190	4298	−1108	0.036	5063	765	0.199
HSPA8, Heat shock cognate 71 kDa protein (C)	5261	4270	991	0.008	6381	2111	0.003
Hadha, Trifunctional enzyme subunit alpha (M)	3294	3920	−625	0.033	3412	−507	0.296
Eci1, Enoyl-CoA delta isomerase 1 (M)	2002	3816	−1814	0	3431	−385	0.241
Idh2, Isocitrate dehydrogenase (M)	2772	3741	−969	0.006	3102	−639	0.039
Gnmt, Glycine N-methyltransferase (C)	2178	3355	−1177	0.013	2893	−462	0.199
HSP90b1, Endoplasmin, GRP94 (ER)	4518	3180	1338	0.002	4079	899	0.029
Hibadh, 3-hydroxyisobutyrate dehydrogenase (M)	1836	2957	−1121	0.003	2399	−558	0.034
Ugt1a1, UDP-glucuronosyltransferase 1 (ER)	1652	2547	−895	0.056	1830	−717	0.121
Dmgdh, Dimethylglycine dehydrogenase (M)	1784	2498	−715	0.028	1965	−533	0.078
Gcdh, Glutaryl-CoA dehydrogenase (M)	1547	2479	−932	0.002	1913	−566	0.131
Suclg2, Succinyl-CoA ligase subunit beta (M)	1404	1971	−567	0.003	1622	−349	0.049
Slc25a20, Carnitine/acylcarnitine carrier protein (M) *	1642	1934	−291	0.391	1613	−321	0.218
Sardh, Sarcosine dehydrogenase (M)	1111	1537	−426	0.04	1380	−157	0.442
Cpt2, Carnitine O-palmitoyltransferase 2 (M)	1044	1480	−436	0.006	1235	−245	0.144
Pygl, Glycogen phosphorylase, liver form (C)*	1500	1333	168	0.394	1457	124	0.568
Dhtkd1, 2-oxoglutarate dehydrogenase E1 (M)	1013	1328	−315	0.02	1098	−230	0.175
Aco2, Aconitate hydratase (M)	944	1325	−380	0.009	1286	−38	0.796
Pdhb, Pyruvate dehydrogenase E1 component subunit beta (M)*	934	1084	−150	0.102	1039	−44	0.723
Acadsb, Short/branched chain specific acyl-CoA dehydrogenase (M)	679	1002	−323	0.001	672	−330	0.001
Pdha1, Pyruvate dehydrogenase E1 component subunit alpha (M)*	681	779	−98	0.162	713	−66	0.281
Psma1-7 ; Psmb1-7, Proteasome 20S subunit (C)*	595	557	38	0.582	584	27	0.706
Alt1, Alanine aminotransferase (C)	134	546	−412	0.005	571	25	0.868
Sec13, Protein SEC13 homolog (C, ER, N)	491	321	171	0.009	404	83	0.118
Gys2, Glycogen [starch] synthase, liver (C)	69	147	−78	0.043	74	−74	0.056
Pdk2, Pyruvate dehydrogenase (acetyl-transferring)] kinase isozyme 2 (M)	63	114	−50	0.034	47	−67	0.011
Pcna, Proliferating cell nuclear antigen (N)	127	70	57	0.046	152	82	0.008
Atg3, Ubiquitin-like-conjugating enzyme ATG3 (C)	94	63	31	0.004	83	20	0.295
Srp54, Signal recognition particle 54 kDa protein (C)	44	32	12	0.02	40	9	0.054
Stat5b, Signal transducer and activator of transcription 5B (C,N)	27	15	11	0.01	21	6	0.083
Pdk4, [Pyruvate dehydrogenase (acetyl-transferring)] kinase isozyme 4 (M)*	1	9	−8	0.098	1	−9	0.071
Igfbp1, Insulin-like growth factor-binding protein 1 (S)	1	6	−5	0.005	0	−6	0.002
Atg7, Ubiquitin-like modifier-activating enzyme ATG7 (C)*	2	3	0	0.745	3	1	0.221
Igfals, Insulin-like growth factor-binding protein complex acid labile subunit (S)	12	3	10	0.014	9	6	0.168
Igf1, Insulin-like growth factor I (S)	55	2	53	0.015	9	7	0.094
Cdk9, Cyclin-dependent kinase 9 (N) *	1	2	−1	0.294	0	−2	0.222
Nr3c1, Glucocorticoid receptor (C,M,N)*	2	1	1	0.395	2	1	0.479
Becn1, Beclin-1 (ER, G, M)	1	0	1	0.081	1	1	0.146
Sqstm1, Sequestosome-1	4	2	−2	0.500	31	28	0.500
Atg2a, Autophagy-related protein 2 homolog A (C) *	0	0	0	0.313	1	1	0.280

Quantities of individual proteins mentioned in text expressed in copy numbers of polypeptide per micron cube of liver cell. Red: Chaperone proteins. Proteins with asterisks have a non-significant variation between RES and AL. Presumed cellular localization: C-cytosol, ER-endoplasmic reticulum, G-Golgi, M-mitochondrion, N-nucleus, S-secreted

## DISCUSSION

We have shown previously and in this study that after ten days of 40 % food restriction (RES), the body weight of the young rats merely increased 1.14 fold, whereas in unrestrictedly fed rats (AL), it increased by over 2.2 fold. Moreover, in a single day of unrestricted re-feeding, CU rats underwent an abrupt 1.3 fold increase of body weight [5] (Table 1). Confirming that the rapid CU effect on weight was not due solely to the food load in the gut, this trend was observed in individual organs, which were more than twice heavier for AL than for RES rats. Similarly, following the single day of re-feeding, a dramatic weight increase of RES rat livers of about 1.7 fold was measured [11]. It should be noted that, as evidenced by the 1.14 fold net weight gain of their whole bodies and livers, and the increase in tibia length [40], the RES-fed rats were clearly not calorie-depleted. Rather, as in nature, they were restricted in their access to excess food and consequently, RES should not be considered as a non-physiological state. In contrast, because unlimited access to food is very rare in nature, AL-treated rats should not be considered as being at a *bona fide* physiological state, AL should not be considered as being the control reference in research on metabolism and liver function. In terms of height gain in youth, AL feeding is probably the preferred mode, as shown by the secular trend of increased human height in most industrialized countries in the past decades [41]. However, there is growing evidence that AL feeding has deleterious effects on adult metabolism, which in the long term might accelerate aging and induce the onset of degenerative diseases, leading to early death [26].

In this study, we focused on the liver as it is most rapidly and profoundly affected by nutritional manipulations. Liver is the main source of insulin like growth factor (IGF)-I and is strongly associated with linear growth in children. Whereas no significant differences in the relative fat content were found among the three groups, expectedly, RES rat livers contained significantly less glycogen than AL livers [11]. Earlier observations in older rats have shown that the livers generally maintained similar amounts of cells in RES, AL and CU [42], implying that the size of RES liver cells was half of the AL cells with minimal levels of glycogen [11]. Yet, we also found that the RES cells accumulated 7 % of new proteins mostly in the mitochondria, to drive the catabolism of carbohydrates, lipids and proteins. This happened alongside an apparent mild across-the-board degradation, or slower replenishment, of the most abundant proteins in the endoplasmic reticulum and the cytosol, among which, the ribosomal proteins and HSP90 and HSC70 chaperones acting as the core components of the protein homeostasis machineries [28].

Quantitative proteomics confirmed earlier data showing a RES-induced mitochondrial biogenesis of oxidative and stress defensive genes and support ATP synthesis in general [43, 20]. In the presence of limiting carbohydrates, the degradation of proteins into toxic ammonia and carbon chains to be further fed into glycolysis and the TCA cycle, implies very active mitochondria. It was thus not unexpected to find CPS1 being the most massively increased protein in RES liver cells. Given that mitochondria occupy about 20 % of the volume of rat liver cells [44], as much as 35 % of total proteins in the mitochondrial stroma could be CPS1. Demonstrating a remarkable flexibility of the mito-chondria to carry various organ-specific functions, CPS1 expression was merely 0.003% of the total protein mass in human HepG2 cells [28].

### Effects of nutritional manipulation on growth factors and metabolic enzymes in the liver

The near-limiting energy balance in RES leads to massive changes in several hormones, which have direct effects on linear growth and on the body size of young RES animals (for review see [12]). Linear growth is predominantly regulated by growth hormone (GH) that affects growth directly upon binding to its receptor (GHR), and indirectly, by way of the insulin like growth factor (IGF-1), which is produced mainly in the liver. IGF-1, is both the main mediator of GH action and a GH-independent growth factor, stimulating cell proliferation and differentiation and protecting cells from apoptosis. GH and IGF-1 concentrations are known to be responsive for the changing of nutritional status: fasting reduces their serum levels in mice and rats [45, 46]. Although at the current limited depth of our proteomic analysis, we could not detect GHR in any sample, the transcription factor STAT5b, which is essential for the signal transduction of GHR was found to be remarkably reduced in RES (Table 2). This suggests that under RES, GHR signaling might be attenuated, and explains the reduced amount of IGF-I produced. Here, quantitative proteomic showed a dramatic reduction in the production of IGF-I in RES livers (Table 2), in agreement with the data observed in the serum of the same animals [47]. A significant increase was found already after one day of re-feeding (CU), although IGF-I levels were still lower than AL, similar to what was found in the serum [47]. IGF-I circulates in the plasma tightly bound to o specific IGF binding proteins (BP) extending the serum half-life of IGF peptides, transporting the IGFs to target cells and modulating IGF interactions with their surface membrane receptors. IGFBP-1 binds IGF-1 with high affinity, inhibiting its binding to the receptor, while IGF-1 suppresses IGFBP-1 protein levels, probably at the transcriptional level [48]. Our results show that IGFBP-1 was practically *de novo* synthesized during RES and its level was reduced upon re-feeding (Table 2), in agreement with previous findings that IGFBP-1 production is significantly increased by RES and dietary restriction of proteins [49] and is suppressed by insulin [50, 51]. In contrast, the level of IGF-ALS, a protein that stabilizes IGF-I in the serum, was found to be reduced in RES (although results did not reach statistical significance, in some RES samples it was undetectable) and increased in CU to a level lower but similar to that of AL (Table 2, Supplementary Table 1). Altogether, these changes indicate a RES-induced decrease of IGF-I, contributing to the growth attenuation.

A near limiting energy balance also leads to increased glucocorticoids (GCs) levels. Numerous clinical, animal and cell cultures studies reported a direct negative effect of GC on growth directly [52], or through their stimulatory effect on hepatic IGFBP-1 synthesis [53]. Cdk9, associated with the GC receptor (GR) [54] was indeed found to be increased in RES samples (although NS), indicating increased GR activity leading to growth inhibition.

Glycogen is the major storage form of glucose to be found mainly in liver and muscles. With up to 10 % of their weight as glycogen, livers of AL-fed animals have the highest specific glycogen content of any other body tissue. As after eleven days of RES regimen, we failed to detect any positive staining for glycogen [11], part of the observed differences in total liver weights could be attributed to the maintaining of the glycogen stores to the lowest levels possible. Glycogen homeostasis involves the concerted regulation of the rate of glycogen synthesis (glyco-genesis) and breakdown (glycogenolysis). These two processes are reciprocally regulated, such that hormones stimulating glyco-genolysis (e.g. glucagon, cortisol, epinephrine, and nor-epinephrine) are simultaneously inhibiting glycogenesis. On the other hand, insulin, which directs the body to store excess carbon for future use, stimulates glycogenesis while simultaneously inhibiting glycogenolysis. The basic metabolic pathways for the catalytic conversion of glycogen involve the action of several enzymes, among which, glycogen synthase (GS) (Gys2) that we found to be significantly upregulated in RES, that catalyzes Glycogen elongation, and glycogen phosphorylase GP (Pyg1) (that was somewhat reduced in RES) that catalyzes its breakdown [55]. A possible explanation for these results may be that glycogen is already present and abundant in the AL livers such that excess glucose needs to be redirected to fatty acid synthesis, resulting in reduced GS in AL and CU conditions. By contrast, in RES, more GS is required to favor glycogen formation in case there is a sudden glucose intake. The ratio between the two suggests that in AL and CU, glycogen catabolism is, when needed, strongly favored, whereas in RES, glycogen catabolism, although much needed is not favored, for lack of glycogen expectedly.

With prolonged near-limiting energy sources in 40 % RES, the primary glucose source is gluconeogenesis, the synthesis of glucose from non-carbohydrate precursors, such as glycerol, lactate and amino acids. One of the major enzymes responsible for this metabolic flexibility is the pyruvate dehydrogenase complex (PDC), a mitochondrial multi-enzyme complex that catalyzes the oxidative decarboxylation of pyruvate. Suppressing PDC activity by pyruvate dehydrogenase (PDH) kinase (PDK) can inhibit conversion of pyruvate to acetyl-CoA, resulting in a shift of pyruvate to the TCA cycle or fatty acid synthesis toward gluconeogenesis [56] as found here. Our data showed a dramatic increase in the levels of PDK4 in RES, whereas it was low or absent from the AL and CU samples. This was in compliance with the increase in gluconeogenesis, and with earlier results on skeletal muscles during energy deprivation [57] and with data showing that transcription of the PDK4 gene is elevated by GCs and inhibited by insulin [58].

Recent studies have shown that several histone deacetylase (HDAC) are increased in livers of RES rats. Unfortunately, our analysis could not detect most of the HDAC proteins, nor those of the Sirtuin class SIRT1 [59], SIRT5 [59-61] or HDAC10 [11] that were found by other methods to be increased in RES. SIRT5, a mitochondrial matrix protein, was previously shown to deacetylate and activate CPS1 [61, 62], suggesting that CPS1 activity may be higher in RES livers both because of increased activity [61] and because of its increased level (current study).

Our previous studies showed high autophagy in RES [11]. Here we could detect Beclin1, Atg2a, Atg3, Atg7 and sequestosome 1 (SQSTM; p62). Interestingly, in most RES livers, the levels of Beclin-1 and Atg2 were reduced below the detection limit. The level of p62 was significantly reduced, in agreement with our previous semi-quantitative observations by western blots. These results support our previous data showing that autophagy occurs in livers of the RES rats, probably through HDAC10 deacetylation of HSP70 [11].

We examined the expression levels of the serine/threonine-protein kinase mTOR, which is crucial for developmental growth. mTOR expression is activated by food and numerous studies have shown high mTOR activity in AL, as compared to RES conditions [63]. Remarkably, upon completion of development, mTOR causes aging and age-related diseases [64]. Here, the naturally low cellular levels of mTOR were at the limit of resolution of the method use. Yet, a coherent 25% increase of mTOR amounts was observed in CU as compared to RES (supplementary Table 1), in agreement with its activity at increasing the translation by more ribosomes of new proteins and for the proper folding of which, increased levels of HSP-chaperones are needed. Additional proteins known to be part of the autophagy complexes [65] could not be detected in the MS results.

Food restriction without malnutrition increases longevity and delays the onset of age-associated disorders in numerous species, from unicellular organisms to laboratory mice, rats and primates [18], and significantly improves age-related and all-cause survival in all tested animals. In young animals, RES leads to growth attenuation, which can be reversible up to a certain point, as the EGP is able to conserve much of its growth capacity until conditions improve, enabling CU growth. The growth restricting conditions also slow down the normal process of senescence, thus keeping the EGP in a “younger phase” until conditions for growth are regained. This delayed senescence hypothesis is supported by a series of elegant studies conducted by Baron and colleagues (reviewed in [66]. To check if a similar phenomenon is also occurring in RES livers, we looked for 107 proteins associated with senescence, of which ten were further validated [67]. Five of these, Stx4, Vamp3, Lancl, Vps26a and Pld3 were quantified, however no significant quantitative changes were observed between the treatments.

Our quantitative proteomic results bring robust arguments to settle an ongoing debate on the effect of RES on mitochondrial proteins [68], as we show strong significant effects both at the qualitative and quantitative levels. Hancock et al. 2011 [68] failed to observe in livers of 30 % RES male Wistar rats, any significant mRNA increase of genes encoding for proteins of the respiratory chain. Indeed the two proteins that were checked in their study (citrate synthase and COX4) and were identified here, remained unaffected by RES, compared to AL. However, in our high-throughput study, numerous other mitochondrial proteins were found to be significantly increased, confirming the results of Nisoli [27].

### The role of molecular chaperones and protein clearance mechanisms in food restriction

In animals, especially in neuronal tissues, cellular aging correlates with increased levels of toxic misfolded protein conformers that can compromise membrane integrity [69], especially of mitochondrial membranes [70], and alter the structure and function of other stress-labile proteins [71]. The primary cause for Huntington, Parkinson, Alzheimer, diabetes type 2 and Creuzfeld-Jajobs diseases, is the accumulation in, and outside cells of various misfolded conformers of specific proteins, such as huntingtin, Ataxin, alfa-synuclein, tau, insulin and PRPsc, respectively [17]. Alongside the accumulation of misfolded proteins in various forms of aggregates, amyloids, fibrils and tangles, reactive oxygen species are also produced: a chronic inflammatory response is observed, causing the onset of programmed cell death [15] and a consequent gradual tissue loss, which can lead to death [16]. Hence, specific mutations in alpha synuclein can greatly increase the propensity of human and model animals to develop Parkinson's disease in youth [72]. Mutations in the PrpC gene can cause the early onset in middle adulthood of fatal insomnia disease, which is a hereditary form of Creutzfeldt–Jacob's disease [73]. Likewise, the longer the polyQ tracts in the huntingtin gene, the sooner the onset of Huntington's chorea [74]. Remarkably, the fact that humans carrying such severe mutations, generally do not develop protein misfolding diseases symptoms during the first decades of their lives strongly indicates that in youth, effective molecular defenses against the accumulation of cytotoxic protein conformers are at work. Yet, by unclear mechanisms, these cellular defenses become increasingly defective once reproductive age is reached [75, 76].

By virtue of their ability to specifically recognize misfolded and aggregated proteins and use ATP to unfold them, the molecular chaperones HSP70s, HSP90s, HSP60s and members from the ubiquitin-proteasome pathway can act to reduce the cellular concentrations of proteotoxic conformers [75]. Thus, a complex network of molecular chaperones, co-chaperones, foldases and proteases (called “chaperome”) may accumulate into being 10 % of the total protein mass of human cells [28, 77], to effectively maintain cellular protein homeostasis in youth and retard beyond reproduction age the onset of hereditary and spontaneous stress-induced degenerative protein misfolding diseases [78]. Further supporting a correlation between cellular youth and high cellular load of molecular chaperones, HSP90s and HSP70s in particular that can unfold misfolded conformers, embryonic tissues, stem cells and cancer cells maintain constitutively high levels of molecular chaperones, compared to terminally differentiated tissues [28]. In contrast, for unclear reasons, normal aging cells gradually fail to maintain sufficient levels of adequately active HSP chaperones and consequently, gradually fail to clear toxic protein aggregates, leading to aging and a shorter life span [75]. Whereas calorie restriction has been shown to inhibit growth and tumorigenesis of B16F10 cells *in vitro* and *in vivo*, higher levels of GFP-tagged Hsp70 were reported in heat-treated and non treated calorie restricted cells, compared to AL cells [79]. Here, in terms of number of molecules per micron cube cell, we also found that AL liver cells contain about 76 less HSPA1A molecules than food restricted cells, a decrease that was, however, counterbalanced by a majority of 990 more HSPA8 molecules, sharing 90 % sequence homology with the minority of HSPA1A molecules (Supplementary Table 1).

Our quantitative data show that the two main ER chaperones HSPA5 (GRP78) and HSP90B1 (GRP94), were lower by about 25-30% in RES livers compared to AL, an effect that was partially reversed in CU (Table 2). This finding is in good agreement with previous mRNA expression data showing in mice RES livers the lower expression GRP78 and GRP94 mRNA [80]. It was proposed that dietary restriction might reduce the extent of protein glycation in the ER lumen, thereby reducing protein misfolding and the need for chaperone assistance, compared to CU and AL livers [80].

### Placing quantitative proteomic data in an ecological/evolutional context

Because in the wild, food is generally limiting, most animals, including humans in pre-agricultural societies, have evolved to thrive when alternating between long periods of mild RES and sustained physical efforts, during which increasingly valuable carbon sources needed to be consumed according to priorities (carbohydrates->lipids->proteins), and short periods of *ad libitum* feeding, during which proteins needed to be rapidly re-synthetized and lipid and carbohydrate stores to be replenished.

This study suggests that it might be inappropriate to consider Ad libitum feeding as a natural physiological state for rodents, and possibly also for humans. Some very young mammals and adults of species that can hibernate, contain brown fat tissues in which special mitochondria can disconnect carbohydrate oxidation from ATP production and thus produce heat from excess fat and carbohydrates [81]. White and beige adipocytes can respond to nutrient scarcity by acquiring a brown-like phenotype with mitochondria expressing oxidative mitochondrial stress defensive proteins, such as super-oxyde dismutase (SOD2) [43]. In agreement, our data also showed that mitochondrial SOD2 was significant higher by 20% in RES as compared to AL. This trend was dramatically reversed during re-feeding (CU), with an observed 27 % decrease of mitochondrial SOD2.

But this mechanism, which is active particularly in very young children, is gradually lost in adulthood [82, 83]. Once lost, such an effective carbohydrate “overflow” safety mechanism, the AL feeding, which is currently a common regimen for young and aging humans with reduced physical activity in industrialized countries, can thus lead to morbid obesity that reduces life span by increasing the risks for metabolic diseases associated to protein misfolding, such as diabetes type II, heart disease and other degenerative diseases [14, 83]. Consequently, our proteomic data might gain from being analyzed from a different point of view: mild continuous RES should be considered as a least stressful near physiological condition for which evolution has optimally programmed adult animals to spend most of their life. Because of the massive protein synthesis and cellular growth that occurs within a very short time, re-feeding should be considered as a very stressful condition for cellular proteostasis, necessitating the synthesis of more ribosomes and many more HSP chaperones, which are general hallmarks of cellular stress [29], to prevent harmful protein misfolding events. Indeed our analysis showed significant higher levels of HSP chaperones in both the CU and AL, as compared to RES (Supplementary Table 4). Corroborating our HSP-derived conclusion of AL being stressful compared to RES, livers from 30 % calorie**-were reported to generate significantly less reactive oxygen species and contain more reduced glutathione and SOD2, than AL rat livers [84].

It should be noted that physiologically, there is a fundamental difference between the CU and the AL states: whereas both involve massive protein synthesis that necessitates high levels of HSP chaperones, in CU, the high levels of chaperones which may be maintained several days thereafter, can keep repairing or degrading toxic misfolded protein species and thus sustain optimal proteostasis and cellular youth. In contrast, during chronic excess feeding as in AL, the high levels of chaperones remain maximally challenged by newly synthetized proteins. In the long term, they may fail to repair or degrade some toxic misfolded species, thereby unleashing the degenerative processes of cellular aging and tissue loss [85,14].

The high similarity of proteomic profile of short term CU and heat stress (Supplementary Table 4), suggest that, as in the case of prolong heat shock, the prolong AL stress might result in a gradual decrease in the efficiency of the HSP-chaperone network, leading in the long term to aging and protein misfolding diseases. This is precisely what is observed in over-weight aging AL fed humans that often develop diabetes type II and for which, restricted feeding can reverse the progression of pre-existing obesity and type II diabetes. This suggests a clinically relevant and feasible dietary intervention to prevent and treat obesity and metabolic disorders [86] and maybe also Alzheimer's disease [87]. AL should thus be considered as a non-physiological stressful condition for which humans and animals were not optimized by evolution. Both intermittent and periodic fasting or increased physical activity [88] can increase lifespan, even when there is little or no overall decrease in calorie intake [89, 90]. It is likely that in modern humans, alternating frequent short fasting periods that would break up the un-healthily prolonged AL periods, would recapitulate in part the natural RES/re-feeding alternating cycles for which they were optimized by evolution, thereby retarding early aging otherwise observed in AL and prolonging human life span. It is thus reasonable to speculate that a life-long cyclic regimes alternating long mild RES and exercise periods, for example in the weekdays, with short CU episodes, for example in weekends [91], would retard human aging and delay the onset of degenerative and metabolic diseases, compared to inactive human adults continuously feed *ad libitum*.

This study shows the power of high throughput quantitative proteomic analysis as a method to address general biological questions. It enables the use of new parameters, such as precise amounts and stoichiometric ratios of various metabolic enzymes and cellular machineries, such as the ribosomes, the chaperones and proteases that control cellular protein homeostasis. On the other hand, many important proteins that were previously shown individually by more sensitive methods to be produced by the liver during RES and CU, could not be detected here, limiting our ability to identify novel growth biomarkers. The use of stable isotope labeling by amino acids in whole animals, combined with immunohistochemistry and classic immunodetection analysis with specific antibodies are therefore still required to obtain a broader picture of the quantitative changes in important but scarce proteins, following biological stimuli.

## MATERIALS AND METHODS

### Animals

Male Sprague–Dawley (SD) rats, 21 days old, were purchased from Harlan (now named Envigo, Rehovot, Israel) and housed individually at the animal care facility of the Felsenstein Medical Research Center for three days before the beginning of the experiment, to accustom to the solitary conditions. One group of animals was sacrificed at the beginning of the experiment (24 days old; baseline controls; n=6). The other rats were then divided into two groups: one was given an unlimited amount of food (complete diet for rats and mice, 3.2 kcal/g, 2018Sc, provided by Teklad, Envigo, USA) (ad libitum group – AL, n=6), and the other was given 60% of the same chow (food-restricted group- RES). The 40% restriction was calculated on the basis of a previous study wherein animals were housed individually and the amount of food consumed each day was measured together with the animal's weight and weight gain [5]. All animals had unlimited access to tap water. The food restriction was maintained for 10 days. At that point, the RES group was further divided into two groups: one was kept food restricted (RES, n=6), and the other was given normal chow ad libitum (that induced catch up growth, hence this group was named CU group; n=6). After 1 day of re-feeding, animals from all three groups were sacrificed by CO_2_ inhalation; the liver was removed, weighed and immediately frozen in liquid nitrogen until analyzed. Part of each liver was stored in 4% formaldehyde and was later used for paraffin sections [11]. Throughout the study, animals were observed daily, and all remained bright, alert and active, with no evidence of any disorder. The experiment was repeated twice and three livers from each group were used. The Tel Aviv University Animal Care Committee approved all procedures.

### Folch method for lipid content determination

Chloroform/methanol in a relative volume of 2:1 was added to 100 mg of homogenized liver tissue. After dispersion, the whole mixture was agitated during 15-20 min on an orbital shaker at room temperature. The homogenate tissue was centrifuged to recover the liquid phase, and then the solvent was transferred to a clean tube, washed with 200 μl water and vortexed for a few seconds to obtain two separate phases. The lower phase was transferred to a new tube and air-dried. The amount of lipid extract that was dried in the tube was weighed and calculated/100 mg of tissue [30].

### Immunohistochemistry

Deparaffinized sections were incubated for 25 min in 3% H_2_O_2_ in methanol to inactivate endogenous peroxidases, blocked with 10% non-immune serum compatible with the second antibody, and incubated with a Proliferating Cell Nuclear Protein (PCNA) specific antibody (CAT#18-0110 Zymed Laboratories Inc, CA 94080, USA). Positive binding was visualized with a biotinylated second antibody and streptavidin-peroxidase conjugated with aminoetyl carbazole as a substrate (Histostatin-SP kit, Zymed Laboratories, Inc.). Counterstaining was performed with hematoxylin.

### Protein purification

Rat livers were homogenized in a radio-immuno-precipitation assay (RIPA) buffer (20Mm Tris-HCl, 150mM NaCl, 1% NP-40, 0.25% Na-deoxycholate), that was supplemented with a protease inhibitor cocktail (Roche, Basel, Switzerland) in a 1:12 ratio. Protein concentration was determined using the Pierce BCA Protein Assay Kit (Thermo Scientific, IL, USA) according to the manufacturer's recommendations. 100μg proteins were analyzed per each sample by 10% sodium dodecyl sulfate (SDS)-polyacrylamide gel electrophoresis (PAGE) followed by fixation (methanol: acetic acid: H_2_0 in a ratio of 50:10:40) and staining with Coomassie Brilliant Blue R-250 solution (Fisher BP101-25).

### Proteome analysis

Protein concentrations were determined accurately by gel electrophoresis, Coomassie blue staining, and densitometry, compared against a pre-quantified standard cell extract. 30 micrograms of each sample were separated by SDS-PAGE on Bis-Tris precast gels (4-12% acrylamide, Novex). Entire lanes were sliced using a gel gridding tool (Gel Company, San Francisco, CA) into 14 fractions, which were digested auto-matically with sequencing grade trypsin (Promega Corp., Madison, WI) on a Biomek 3000 liquid handler (Beckman) according to standard protocols [31]. Extracted tryptic peptides were dried and re-suspended in 20 μl 0.1% formic acid, 2% (v/v) acetonitrile for LC-MS/MS.

### Mass spectrometry analysis

Tryptic peptide mixtures were analyzed on a Q-Exactive Plus quadrupole orbitrap instrument (Thermo Fisher, Bremen, Germany) interfaced via a nanospray source to a Dionex RSLC 3000 nano HPLC system (Dionex, Sunnyvale, CA, USA). Peptides from all fractions were separated on a C18 reversed-phase Easy Spray PepMap nanocolumn (50 cm x 75 μm ID, 2 μm) at 0.3 μl/min with a gradient from 4 to 86 % acetonitrile in 0.1 % formic acid in 40 min. Full MS survey scans were performed at 70,000 resolution. In data-dependent acquisition controlled by Xcalibur software (Thermo Fisher), the 10 most intense multiply charged precursor ions detected in the full MS survey scan were selected for higher energy collision-induced dissociation (HCD, normalized collision energy NCE=27%) and fragment analysis in the orbitrap at 17,500 resolution. The window for precursor isolation was of 1.6 m/z units around the precursor and selected fragments were excluded for 60s from further analysis.

### Data analysis and statistics

A total of eighteen rat livers were sampled for MS analysis: six biological replicates of the three different treatments: AL, RES, and CU. This enabled us to obtain statistically significant mean values for a high number of proteins, summing up to be at least 92% of the total protein mass of the cells. For each identified protein, raw LFQ values were normalized by the number of their specific peptide identifiers to obtain an average iBAQ intensity value for each identified polypeptide. We collected iBAQ intensity values for 5138 different proteins, which were distinctly identified at least once in the eighteen biological samples: six RES, six AL and six CU (Supplementary Table 1, Sheet 1). Being the most abundant proteins, their sum was considered as a good approximation of the total protein mass in a given sample [29]. The over five thousand normalized iBAQ values in a given individual sample were thus summed up to produce an estimate of the total (100%) protein mass in that sample. The normalized iBAQ value of each particular protein in a given sample was then converted into a fraction from the sum of the normalized iBAQ signals in that same sample, which was equivalent to the mass fraction value for that protein [28, 29]. For every given protein, a mean of six relative mass fractions was obtained for the six biological replicates of the three treatments (AL, RES or CU) and the extent of variation around the mean was analyzed by t-statistics, hence by obtaining a ratio for the departure of this estimated mean from its notional value and its standard error (Supplementary Table 1, Sheet 2). MS data were processed for protein identification and label-free quantitation with MaxQuant version 1.5.1.2 [32] as described [33] using the set of rat proteome sequences annotated in the SWISSPROT database (RAT.fasta, October 2014 version, 28902 sequences). Both peptide and protein FDR rates were set at 1% as calculated against a decoy sequence database and the match between runs function was activated for LFQ quantitation. Data are available via ProteomeXchange with identifier PXD004068.

Body weight and tissue quantities were expressed as mean ± SD. A one-way analysis of variance (ANOVA) was used to test for significant differences in variables across the three (AL, RES, CU) groups. When differences were found, t-tests were used to ascertain the location of the difference. The significance level was set at p< 0.05.

## SUPPLEMENTAL DATA FIGURES AND FIGURES


