# Supplementary material for: Quantitative proteomics of rat livers shows that unrestricted feeding is stressful for proteostasis with implications on life span
Source: Aging (Albany NY). 2016 Aug 9;8(8):1735–53. doi: 10.18632/aging.101009 (PMC5032693; doi:10.18632/aging.101009)
Supplement: Supplementary file 1 [file aging-08-1735-s001.pdf]

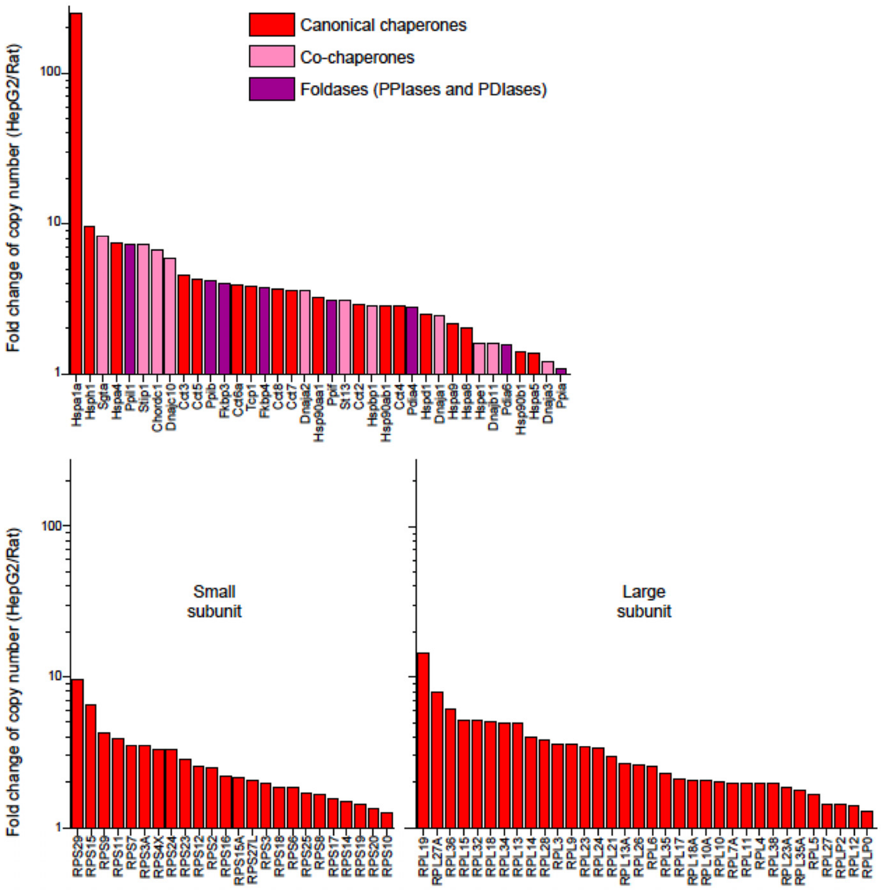

Supplementary Figure 1. Fold difference comparison (in log scale) between human HepG2 cells and rat liver cells of chaperone, co-chaperones and foldases (upper panel) and of ribosomal protein copy numbers (lower panel) per micron cube of cell.

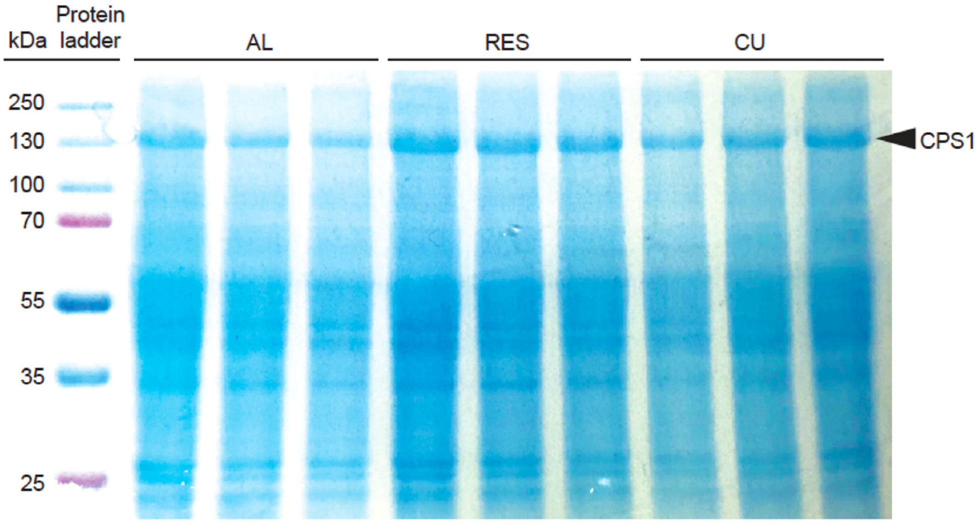

Supplementary Figure 2. Coomassie stained SDS-PAGE of total liver proteins from the AL, RES and CU tissues. Arrow indicates the 165 kDa CPS1.

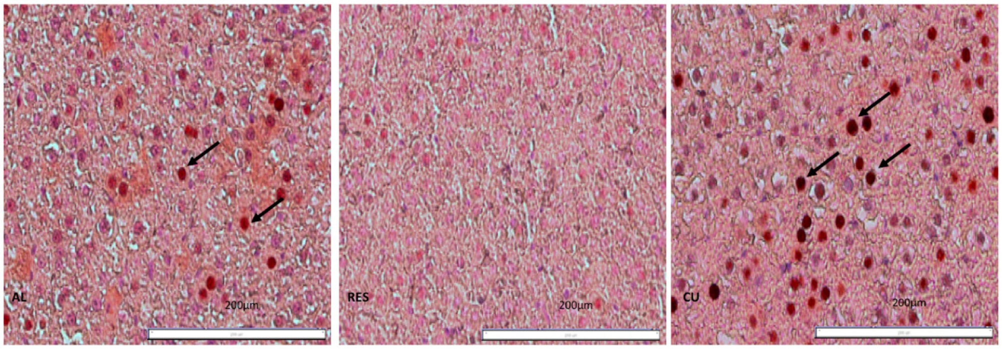

Supplementary Figure 3. Immuno-histochemical staining with peroxidase of AL, RES and CU liver thin sections labeled with specific antibodies against the Proliferating Cell Nuclear Protein (PCNA).

Supplementary Table 1. List of 5138 detected polypeptides in AL, RES and CU rat livers. **Sheet 1**: Raw iBAQs from 18 samples: 6 AL, 6 RES and 6 CU biological repeats. Proteins were sorted alphabetically. **Sheet 2**: Relative mass factions, expressed in % of total protein mass in each of the 18 samples. Proteins were sorted alphabetically. **Sheet 3**: Relative copy number per micron cube cell in the 18 samples. Proteins were sorted alphabetically. **Sheet 4**: Mean mass factions of three AL, RES and CU means and their p-values expressed in % of total proteins. Proteins were sorted from the highest to lowest concentrations in RES. Bold numbers are significant means with P values < 0.05. **Sheet 5**: Mean copy numbers per cubic micrometers cell of AL, RES and CU and their SD. Proteins were sorted from the highest to lowest number in RES.
Please browse Full text version of this manuscript to see this table in Excel format.

Supplementary Table 2A. Significantly quantified proteins from AL-rat liver cells (this study) and human HepG2 cell [33] were sorted according to gene ontology (GO) categories and analyzed for their statistically significant representation in the sample (p<0.05). 21 GO categories were retained for containing each at least 2% of the total cellular protein mass.

PANTHER GO-Slim Biological Process	SUM mass-rat liver (%)	SUM mass-HepG2 (%)
Metabolic process	69.5	63.7
Primary metabolic process	55.6	56.4
Cellular amino acid metabolic process	18.0	5.2
Protein metabolic process	16.7	32.1
Nitrogen compound metabolic process	14.5	6.3
Lipid metabolic process	13.4	5.9
Generation of precursor metabolites and energy	12.9	6.0
Transport	12.4	12.6
Carbohydrate metabolic process	10.6	7.7
Respiratory electron transport chain	8.5	2.5
Fatty acid metabolic process	7.9	2.7
Biological regulation	5.8	11.1
Translation	5.8	12.2
Monosaccharide metabolic process	5.2	3.8
Cellular component organization or biogenesis	4.5	9.8
Regulation of biological process	4.2	8.4
Protein transport	3.7	7.3
Intracellular protein transport	3.6	7.2
System process	3.0	3.8
Multicellular organismal process	2.8	4.1
Proteolysis	2.3	3.6

Supplementary Table 2B. Compared mass fractions of the MS quantified ribosomal proteins from AL-rat liver cells and human HepG2 cell.

Ribosome	Mass-rat liver (%)	Mass-HepG2 (%)
Rps10	0.03	0.04
Rps11	0.03	0.12
Rps12	0.04	0.11
Rps13	0.05	0.06
Rps14	0.06	0.09
Rps15	0.00	0.02
Rps15a	0.03	0.06
Rps16	0.05	0.11
Rps17	0.03	0.05
Rps18	0.06	0.11
Rps19	0.04	0.06
Rps2	0.03	0.10
Rps20	0.07	0.10
Rps21	0.02	0.02
Rps23	0.01	0.04
Rps24	0.01	0.04
Rps25	0.06	0.10
Rps26	0.03	0.03
Rps27	0.01	0.00
Rps27l	0.01	0.01
Rps28	0.01	0.04
Rps29	0.00	0.01
Rps3	0.07	0.14
Rps30	0.01	0.03
Rps3a	0.05	0.17
Rps4x	0.05	0.15
Rps5	0.04	0.09
Rps6	0.06	0.11
Rps7	0.03	0.09
Rps8	0.11	0.19
Rps9	0.04	0.16
RpsA	0.15	0.15
Rpl10	0.02	0.04
Rpl10A	0.06	0.11
Rpl11	0.04	0.08
Rpl12	0.05	0.08
Rpl13	0.03	0.15
Rpl13A	0.03	0.09
Rpl14	0.03	0.10
Rpl15	0.02	0.08
Rpl17	0.02	0.05
Rpl18	0.03	0.15

Supplementary Table 2B. continue

Rpl18A	0.02	0.05
Rpl19	0.01	0.11
Rpl21	0.01	0.04
Rpl22	0.03	0.10
Rpl23	0.02	0.07
Rpl23A	0.05	0.10
Rpl24	0.02	0.07
Rpl26	0.02	0.05
Rpl27	0.07	0.10
Rpl27A	0.02	0.13
Rpl28	0.01	0.03
Rpl29	0.00	0.03
RPl3	0.05	0.19
Rpl30	0.05	0.08
Rpl31	0.02	0.05
Rpl32	0.01	0.07
Rpl34	0.01	0.05
Rpl35	0.04	0.09
Rpl35A	0.01	0.01
Rpl36	0.01	0.07
Rpl36A	0.00	0.01
Rpl37A	0.01	0.02
Rpl38	0.02	0.04
Rpl39	0.01	0.01
Rpl4	0.11	0.22
Rpl5	0.07	0.11
Rpl6	0.06	0.16
Rpl7	0.09	0.15
Rpl7A	0.10	0.19
Rpl8	0.03	0.10
Rpl9	0.02	0.09

Supplementary Table 2C. Compared mass fractions of the MS quantified chaperone proteins from AL-rat liver cells and human HepG2 cell.

Chaperome	Mass-rat liver (%)	Mass-HepG2 (%)
Hsp90ab1	0.61	1.73
Hspa5	0.60	0.83
Hspd1	0.48	1.20
Hsp90b1	0.44	0.61
Hspa8	0.39	0.77
Hspa9	0.18	0.38
Hsp90aa1	0.15	0.57
Hspe1	0.12	0.19
Cct2	0.05	0.14
Cct4	0.04	0.12
Cct3	0.04	0.20
Cct8	0.04	0.14
Cct6a	0.04	0.15
Cct5	0.04	0.16
Cct1	0.03	0.13
Cct7	0.03	0.12
St13	0.03	0.10
Dnajc3	0.03	0.02
Hspa4	0.02	0.19
Stip1	0.02	0.17
Hsph1	0.01	0.13

Supplementary Table 2D. Calculations to establish the volume of a single hepatocyte cell according to Sohlenius-Sternbeck AK; Toxicol In Vitro. 2006; 20:1582-6.

Rat	Value
Net protein mass per gram of liver tissue	112 mg
Liver density	1.05 ± 0.05 g/ml
Volume of 1 g of liver tissue	0.95238 ml
Protein concentration in the liver	117.6 mg/ml
Protein concentration in hepatocyte suspension	0.985 ± 0.211 mg/million cells
Protein mass per hepatocyte in suspension	985 pg/ cell
Mass protein in 100 million cells	98.5 micograms
Number of cells in a microgram of liver	114
Mass of a single hepatocyte cell	8.79464 ng
**Volume of a single hepatocyte cell**	**8354 micrometer cube**

Supplementary Table 3. Sheet 1: 618 proteins that change significantly between AL and RES. **Sheet 2:** 513 Proteins that change significantly between CU and RES.
Please browse Full text version of this manuscript to see this table in Excel format.

Supplementary Table 4. Members of the HSP-chaperome are hallmarks of cellular stresses. Net increased concentrations of eight members of the HSP chaperome, expressed in copy number per micron cube of cell, in human Jurkat cells following a 4 hours heat-shock (HS) at 41°C (from Finka et al. 2016), or in rat liver cells (constantly at 37°C), following AL or CU feeding, compared to the RES regimen.

Protein name	DELTA-MEAN HS	DELTA-MEAN AL-RES	DELTA-MEAN CU-RES
HSPA8	1391	992	2109
HSP90AB1	322	2175	2349
HSPA5	222	2078	1272
STIP1	144	76	135
FKBP4	93	176	226
CCT4	74	246	253
HSP90B1	74	1340	899
DNAJA1	72	85	107

Reference:

Sohlenius-Sternbeck AK. Determination of the hepatocellularity number for human, dog, rabbit, rat and mouse livers from protein concentration measurements. Toxicol In Vitro 2006; 20:1582-1586.

Finka A, Sood V, Quadroni M, Rios PL, Goloubinoff P. Quantitative proteomics of heat-treated human cells show an across-the-board mild depletion of housekeeping proteins to massively accumulate few HSPs. Cell Stress Chaperones. 2015; 20:605–20.
